# Supplementary material for: Genome-Wide CRISPR Screen Identifies KEAP1 Perturbation as a Vulnerability of ARID1A-Deficient Cells
Source: Cancers (Basel). 2024 Aug 24;16(17):2949. doi: 10.3390/cancers16172949 (PMC11394604; doi:10.3390/cancers16172949)
Supplement: Supplementary file 1 [file cancers-16-02949-s001.zip › Supplemental Figure S5_final (raw blot images).pdf]

Genome-wide CRISPR screen identifies KEAP1 perturbation as a vulnerability of ARID1A-deficient cells

Original Blots

Figure S3E

ARID1A

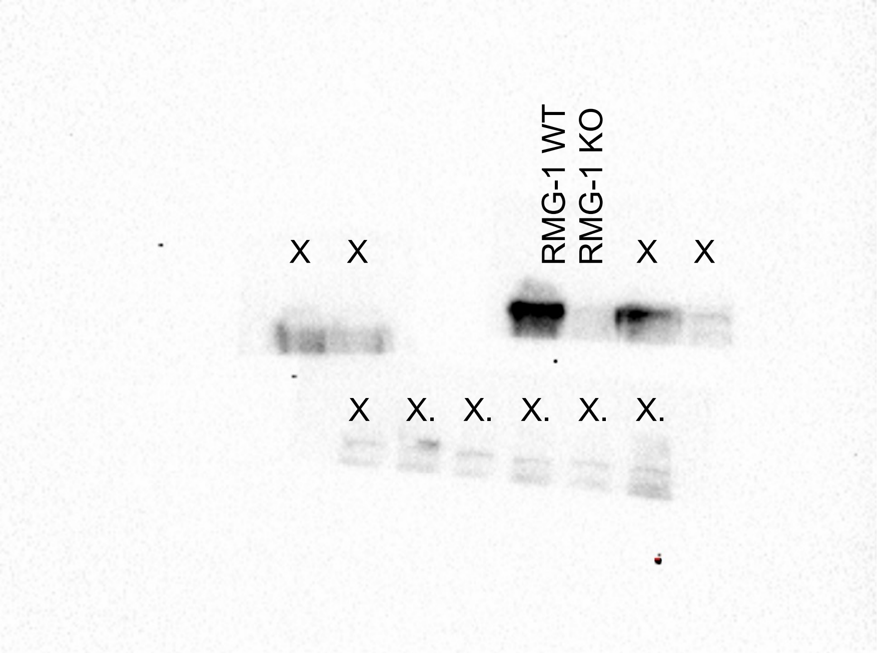

KEAP1

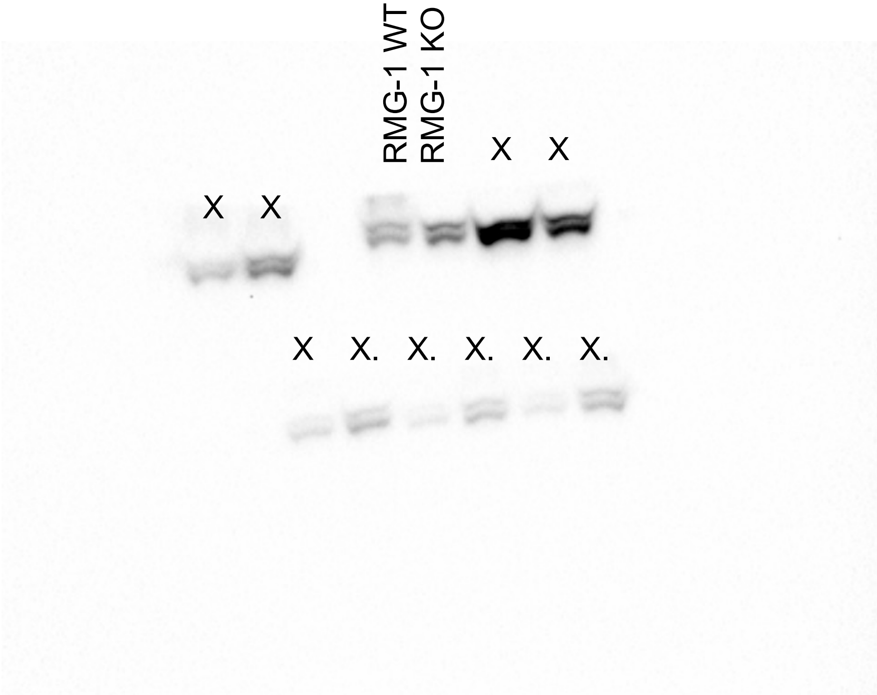

GAPDH

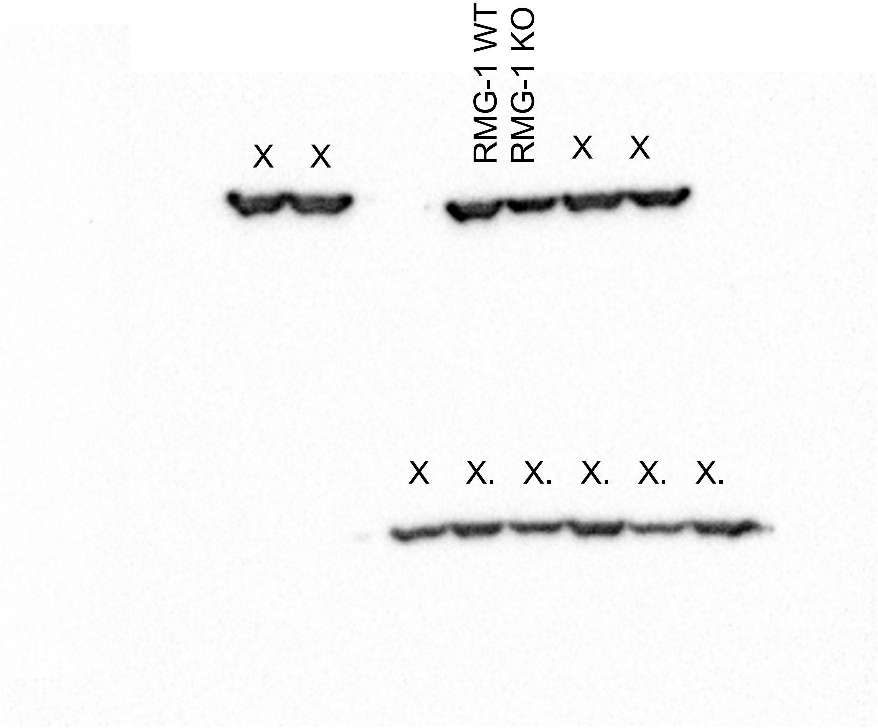

X = not the gel/sample of interest.

Figure S1A

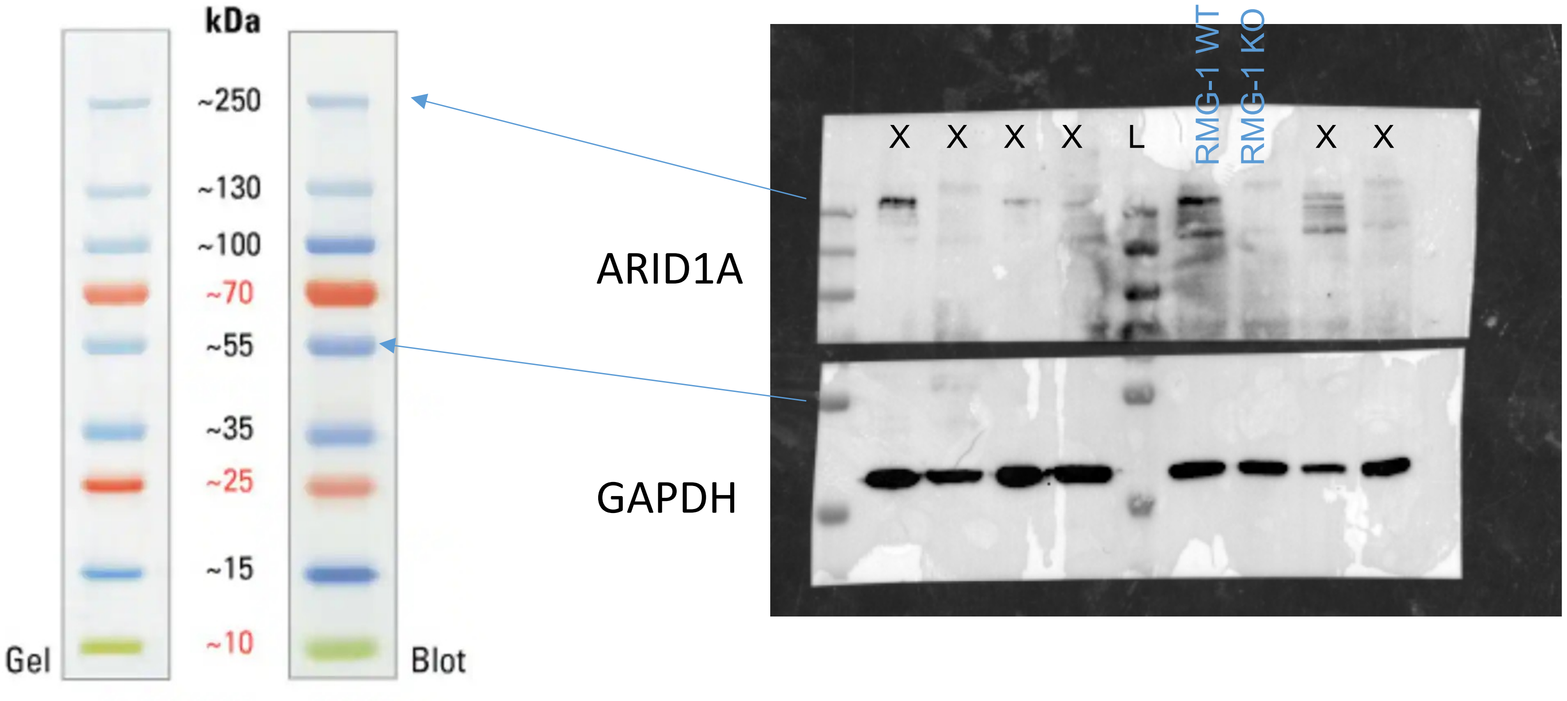

X = not the gel/sample of interest.

Figure S2A

KEAP1 & NRF2

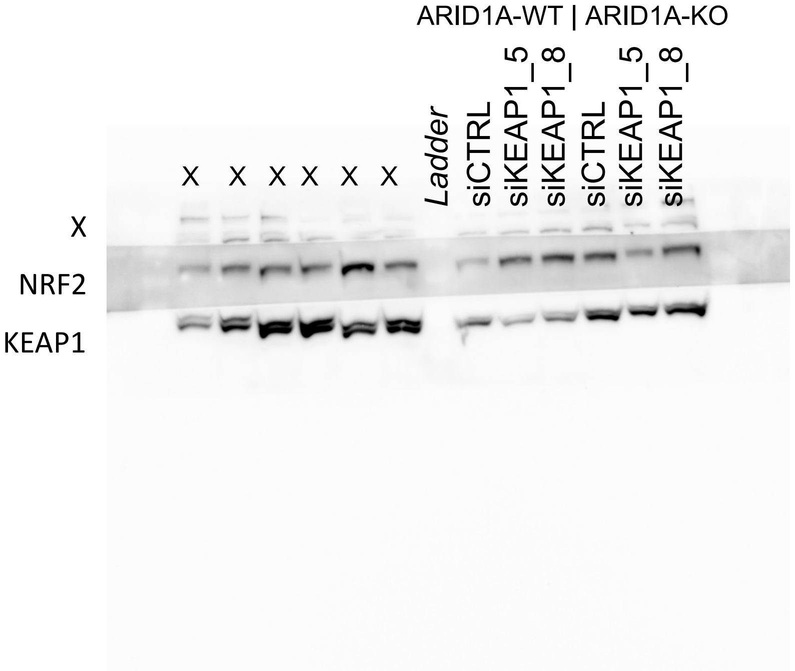

GAPDH

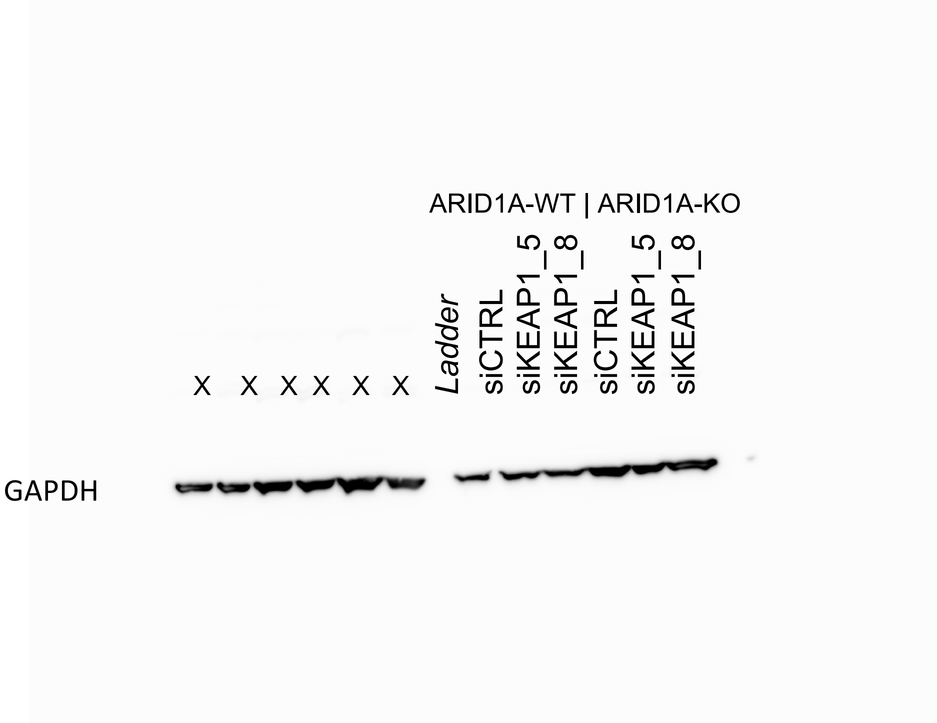

X = not the gel/sample of interest.

Figure S2C

ARID1A

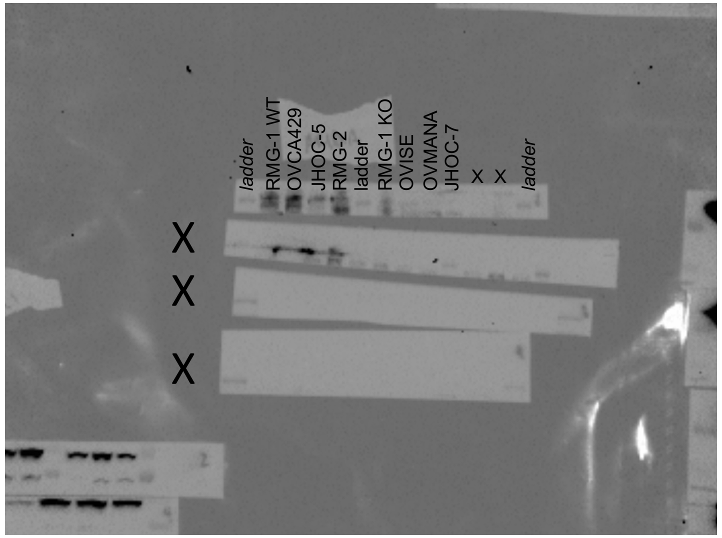

NRF2

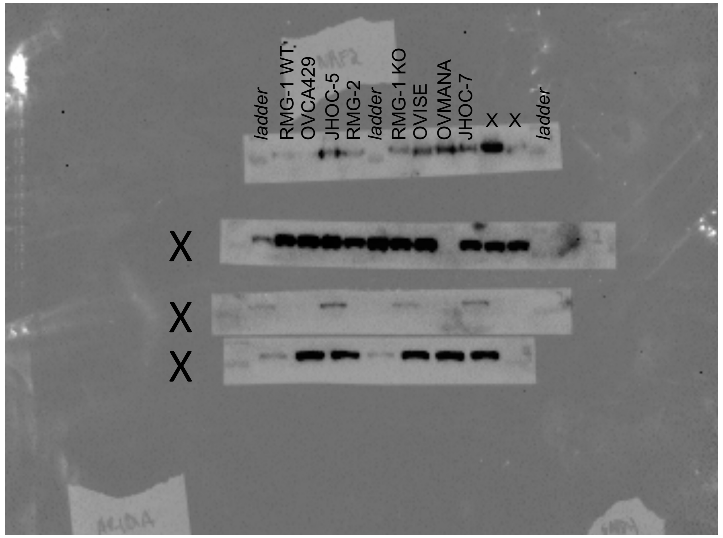

KEAP1

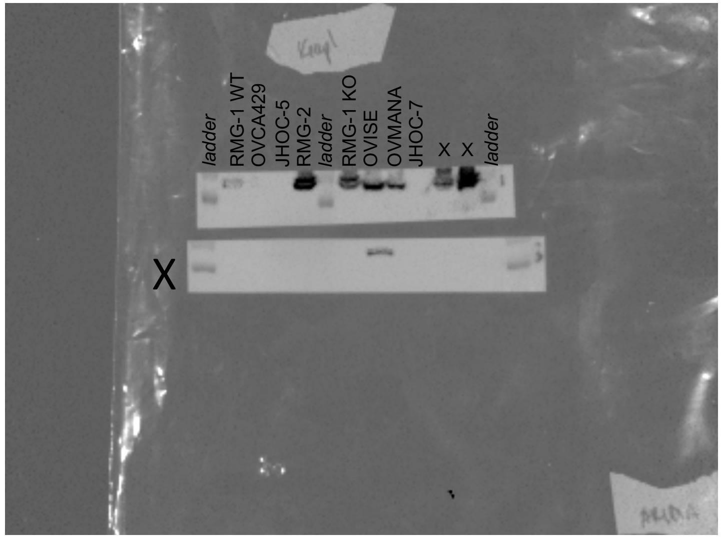

GAPDH

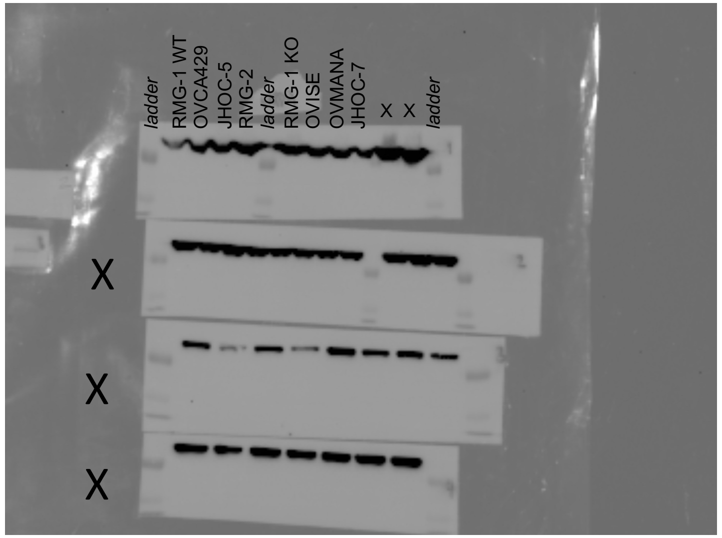

X = not the gel/sample of interest.

Figure S2E

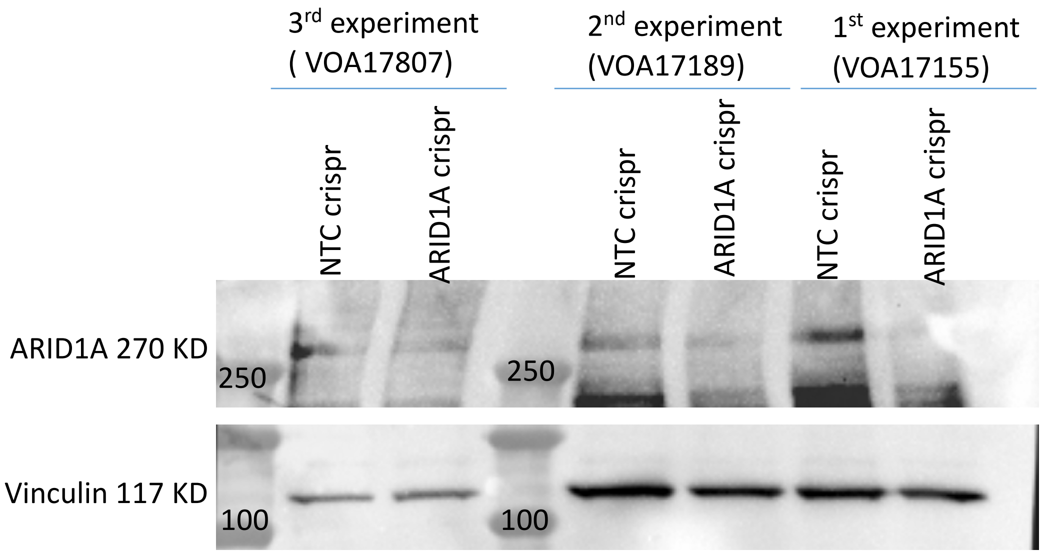
3rd experiment
(VOA17807)
2nd experiment
(VOA17189)
1st experiment
(VOA17155)
NTC crispr
ARID1A crispr
NTC crispr
ARID1A crispr
NTC crispr
ARID1A crispr
ARID1A 270 KD
250
250
Vinculin 117 KD
100
100

Figure S3C

HA

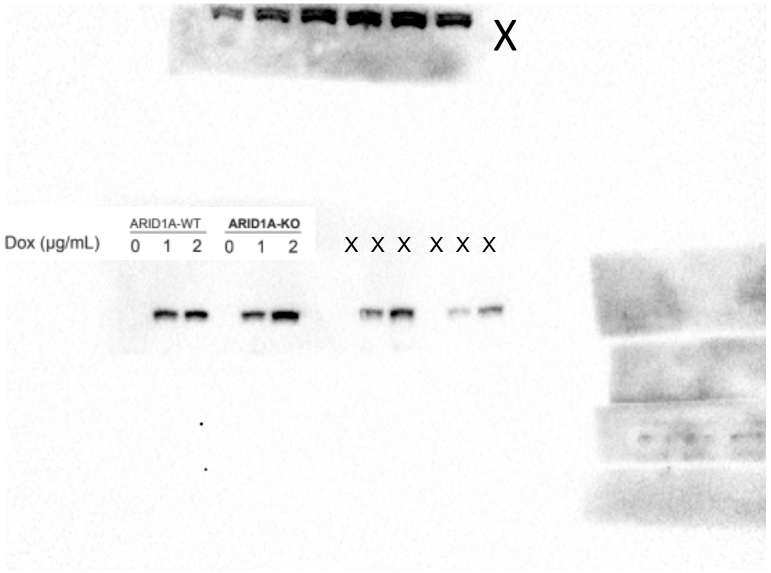

KEAP1

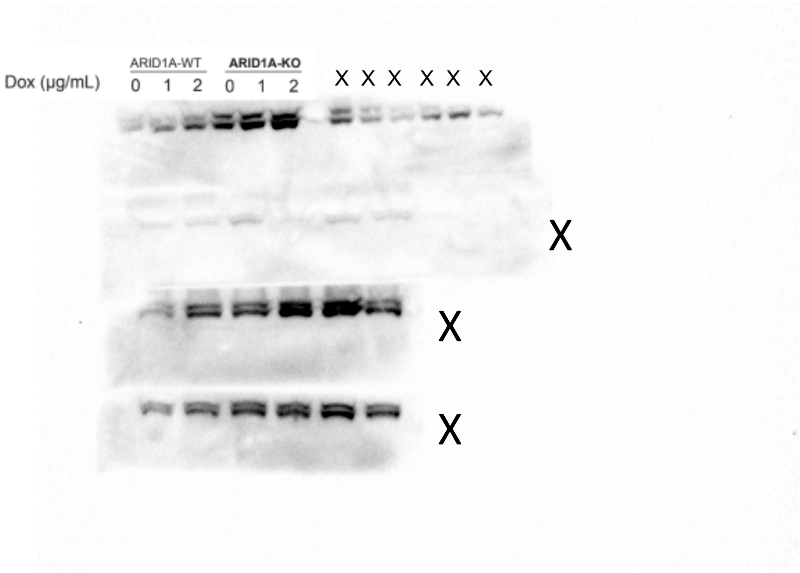

GAPDH

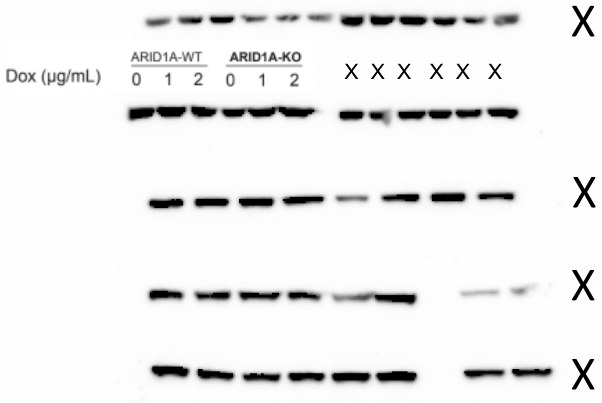

X = not the gel/sample of interest.

Figure S3F

NRF2

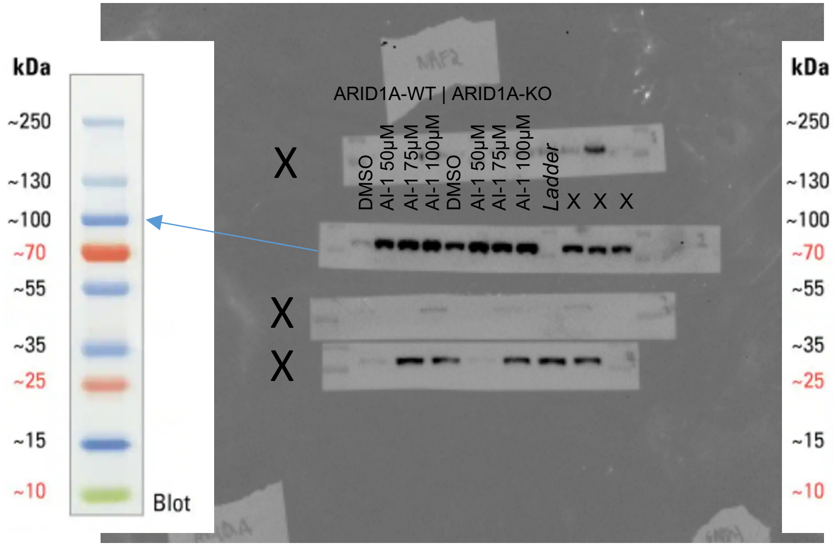

HDAC1

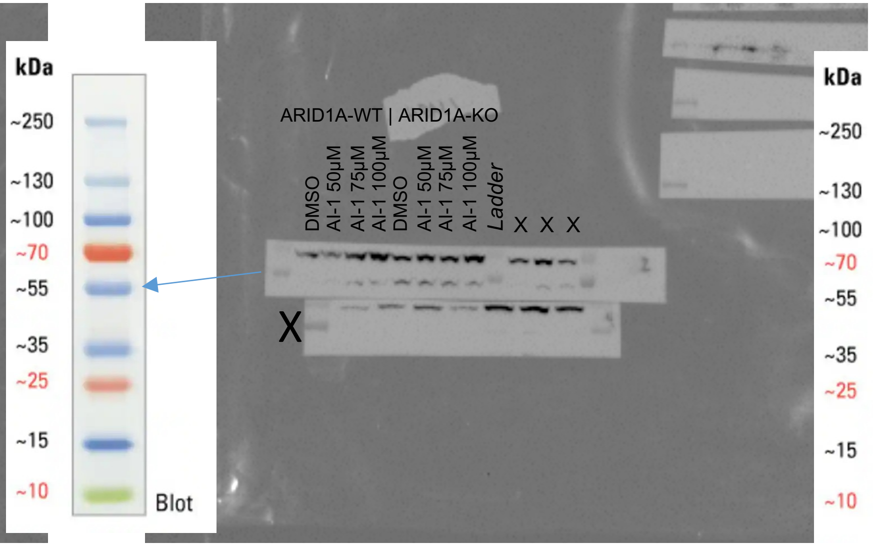

GAPDH

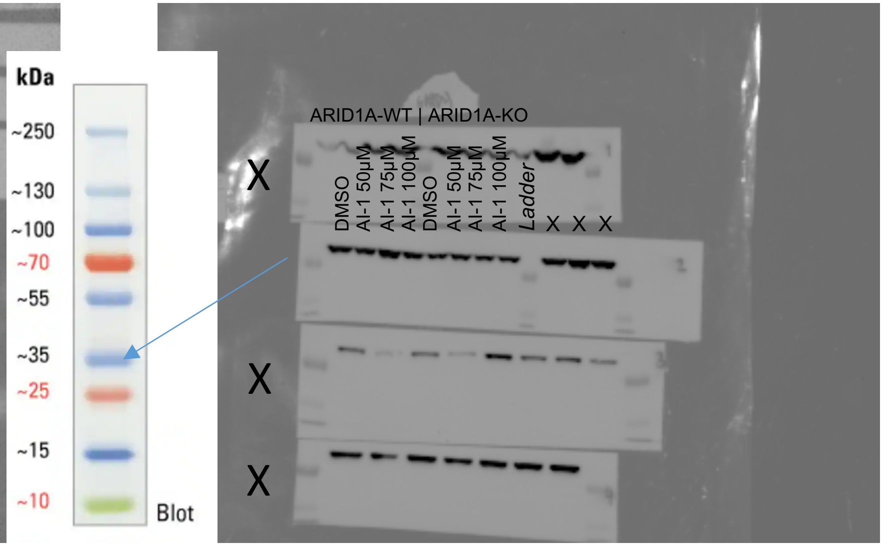

X = not the gel/sample of interest.

Figure S4A

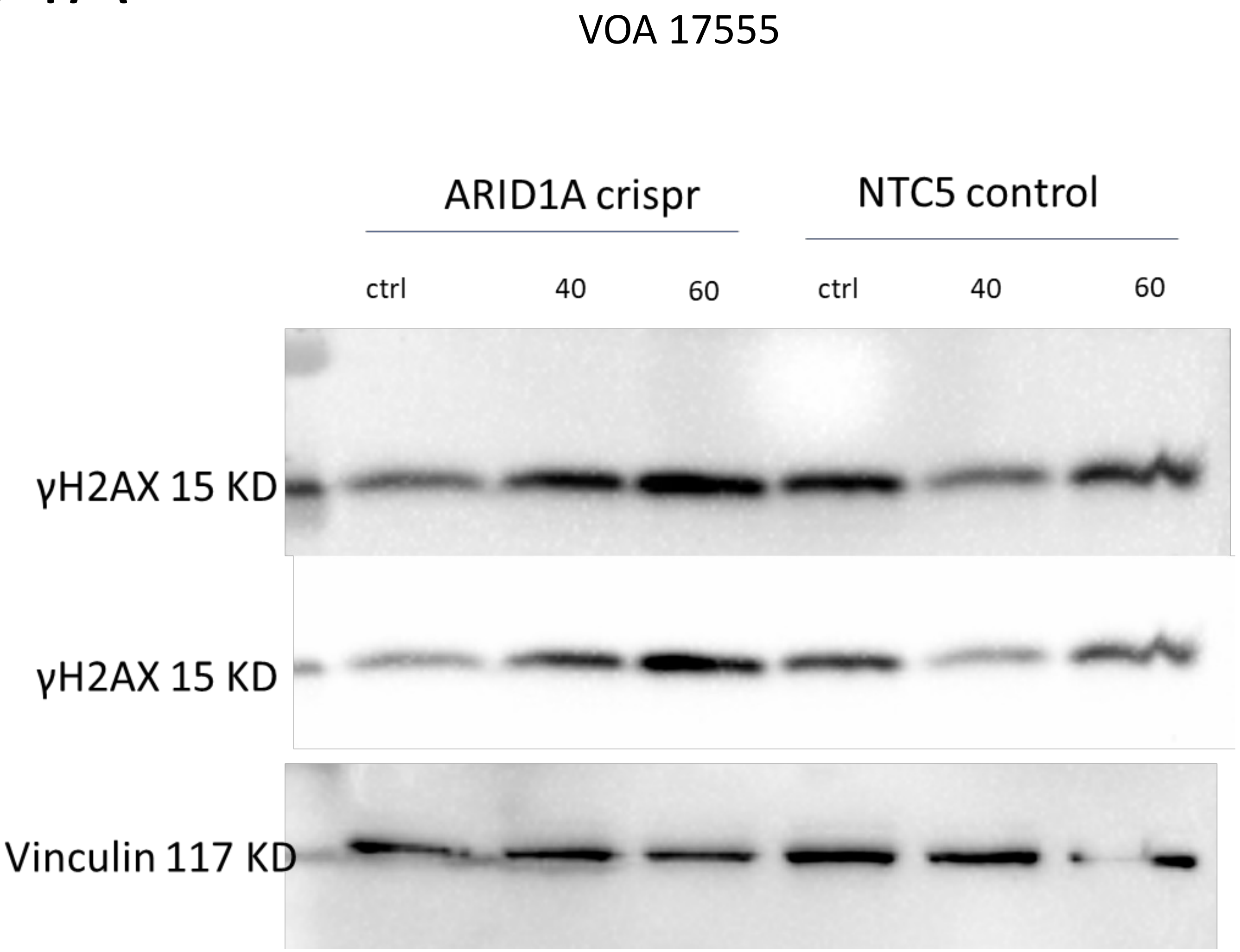
VOA 17555
ARID1A crispr
NTC5 control
ctrl
40
60
ctrl
40
60
γH2AX 15 KD
γH2AX 15 KD
Vinculin 117 KD

Appendix

Additional images

Figure S2C

ARID1A

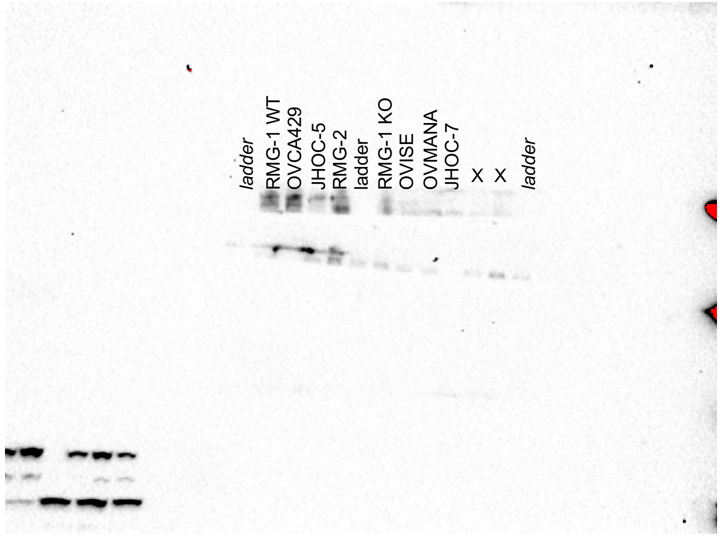

NRF2

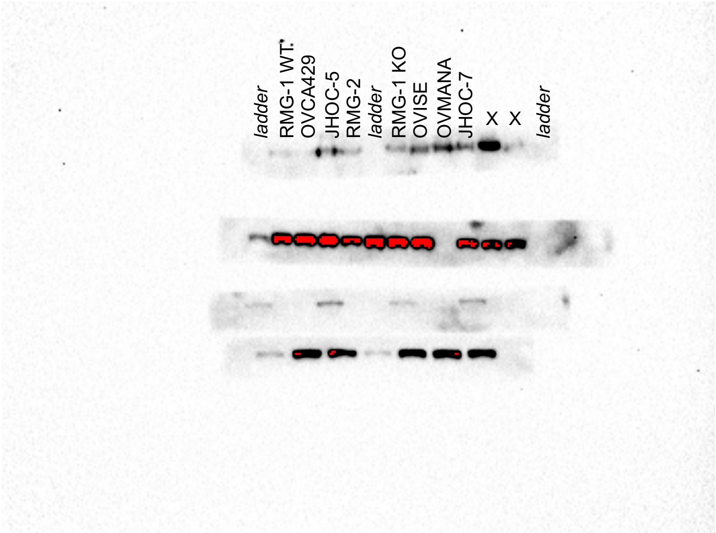

KEAP1

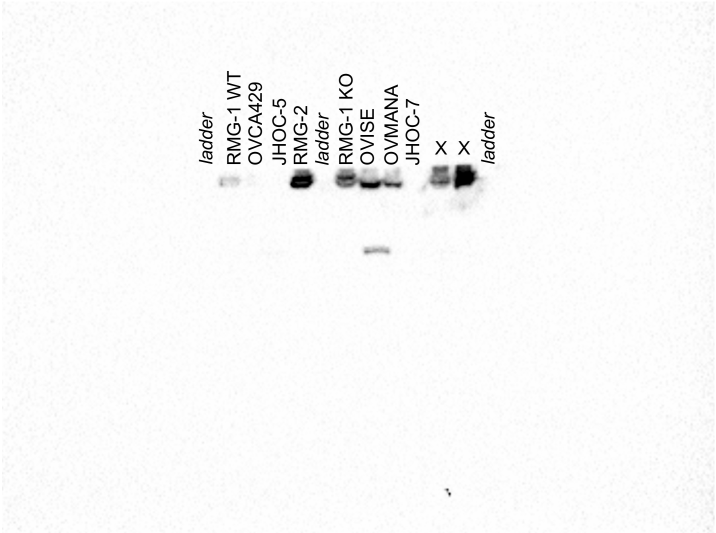

GAPDH

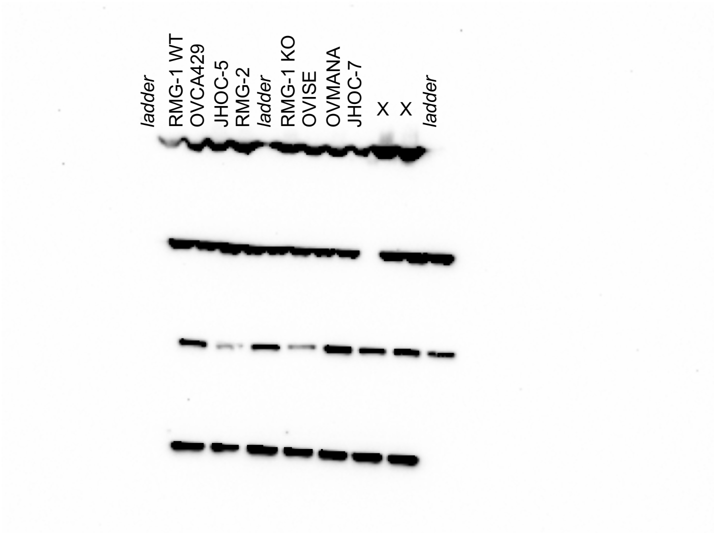

X = not the gel/sample of interest.

Figure S3F

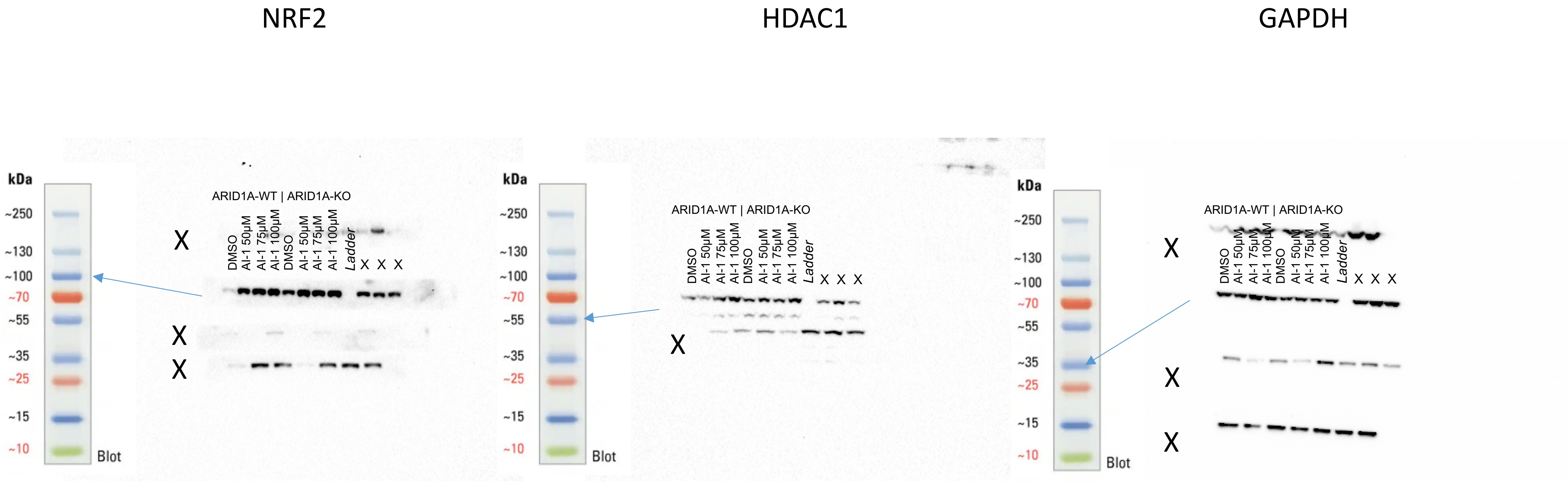

X = not the gel/sample of interest.
