# Supplementary figures and images for: Genome-Wide CRISPR Screen Identifies KEAP1 Perturbation as a Vulnerability of ARID1A-Deficient Cells
Source: Cancers (Basel). 2024 Aug 24;16(17):2949. doi: 10.3390/cancers16172949 (PMC11394604; doi:10.3390/cancers16172949)

Supplemental Figure S1 A

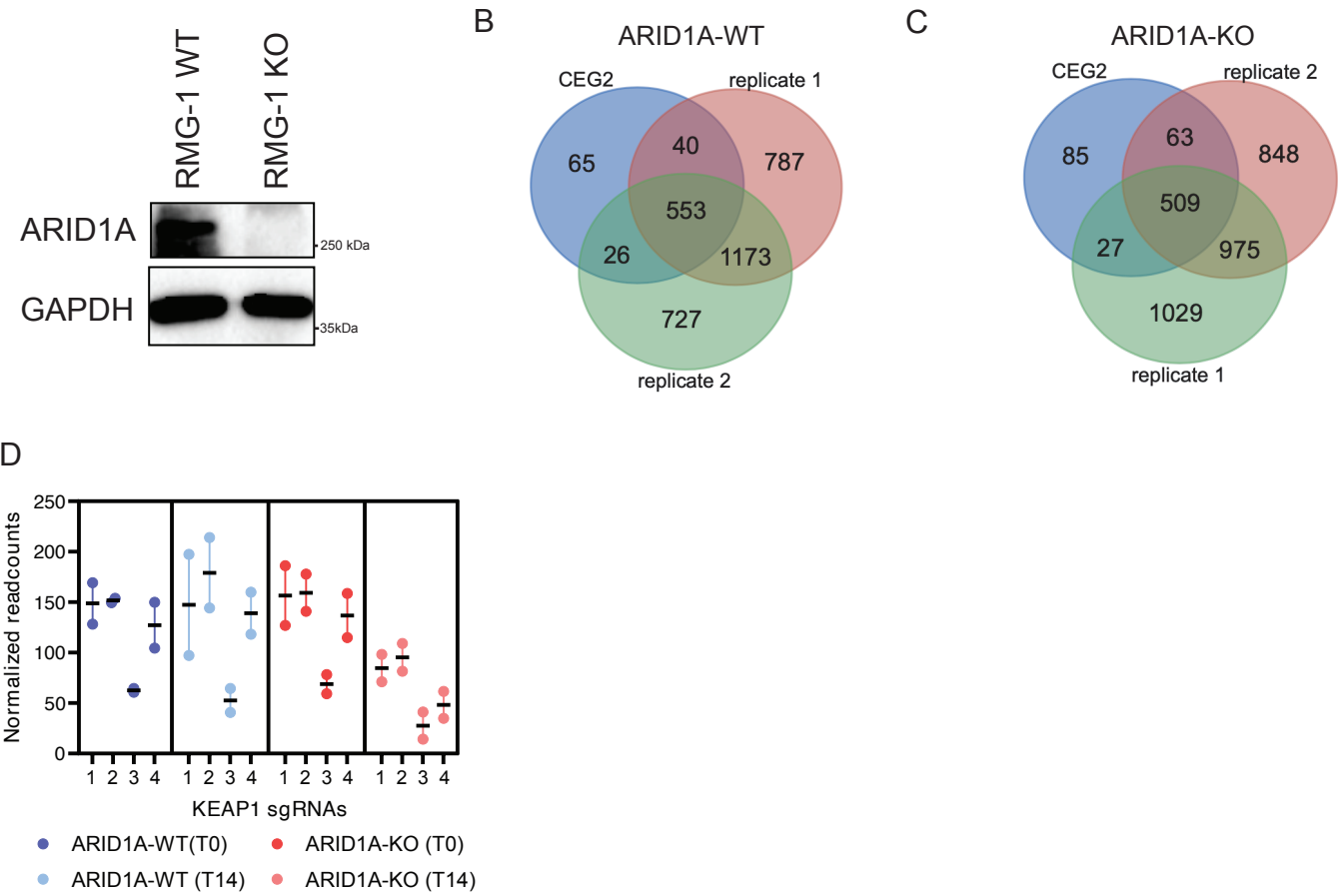

Supplement: Supplementary file 1 [file cancers-16-02949-s001.zip › Supplemental Figure S1_Final.pdf]

Supplemental Figure S2

**A**

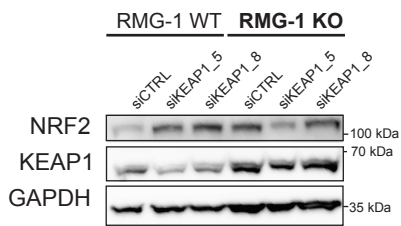

**B**

Cell growth (AI-1)

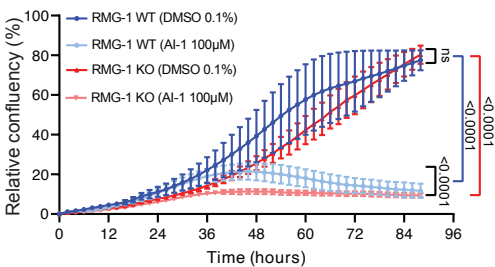

**C**

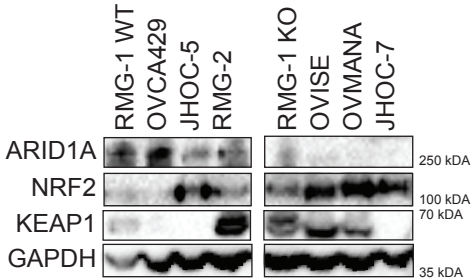

**D**

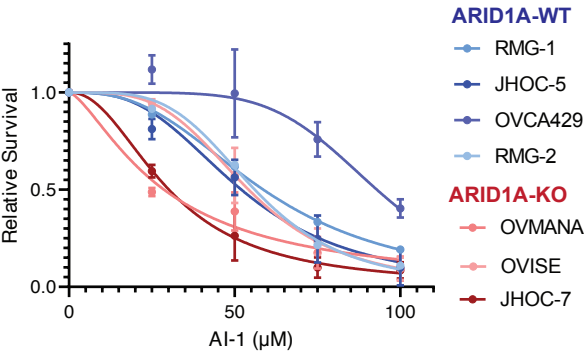

**E**

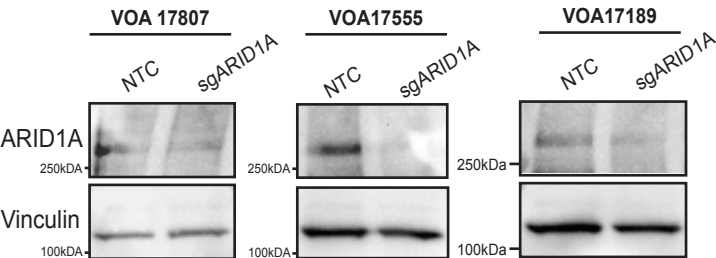

**F**

VOA 17189

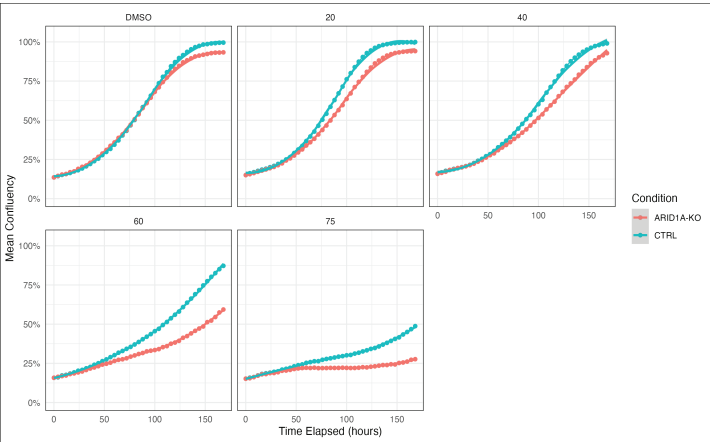

**G**

VOA 17807

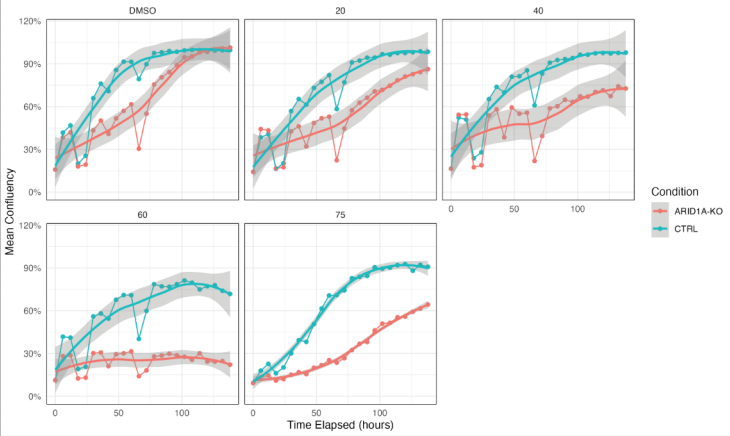

**H**

VOA 17555

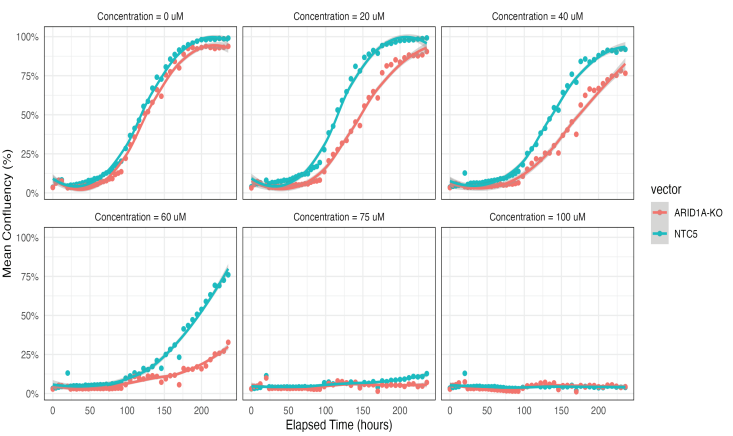

Supplement: Supplementary file 1 [file cancers-16-02949-s001.zip › Supplemental Figure S2_final-2.pdf]

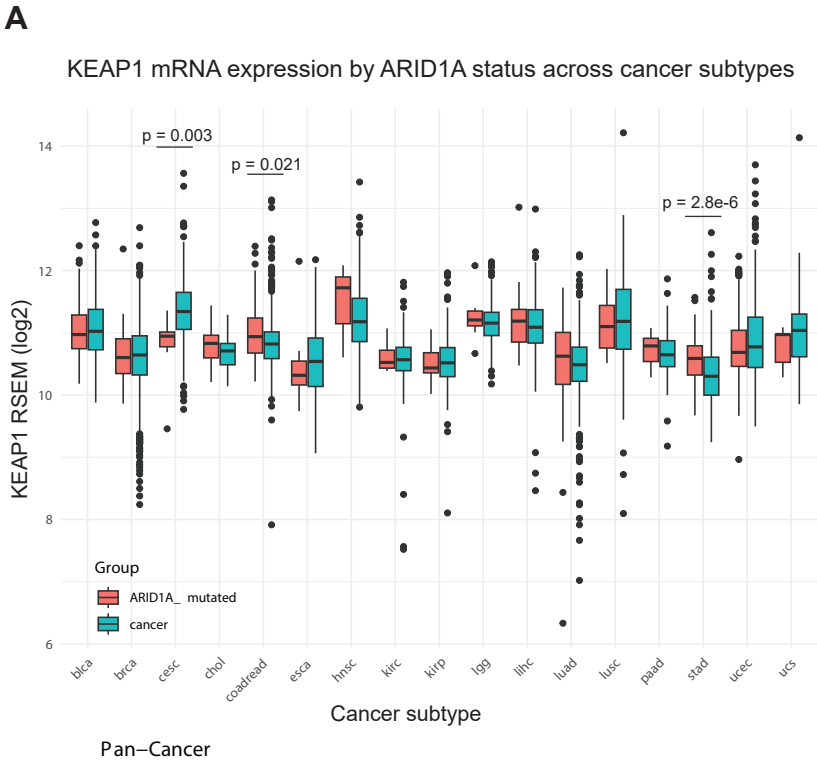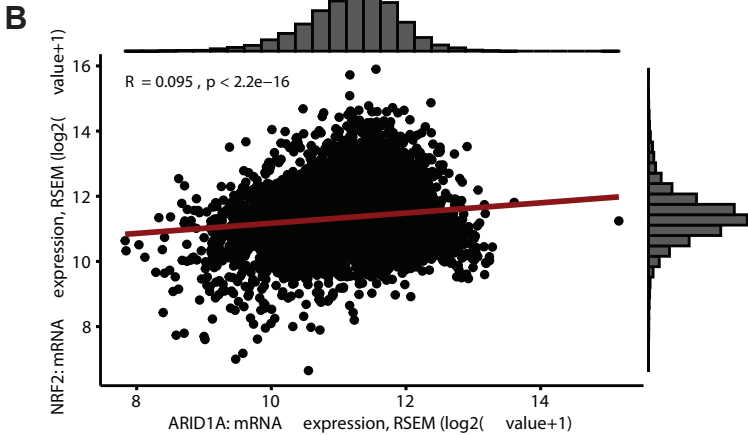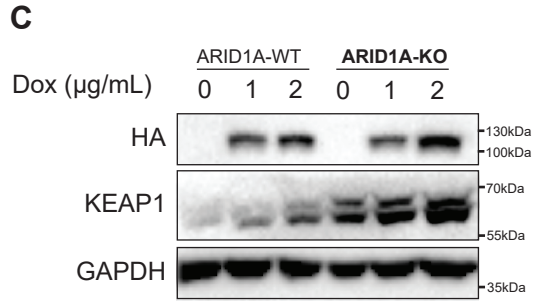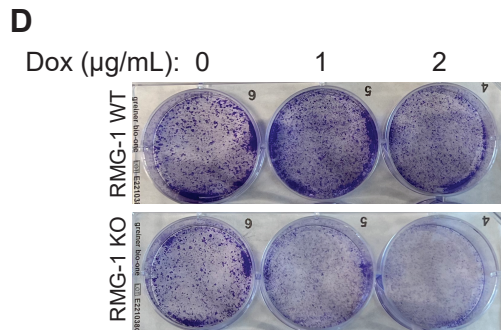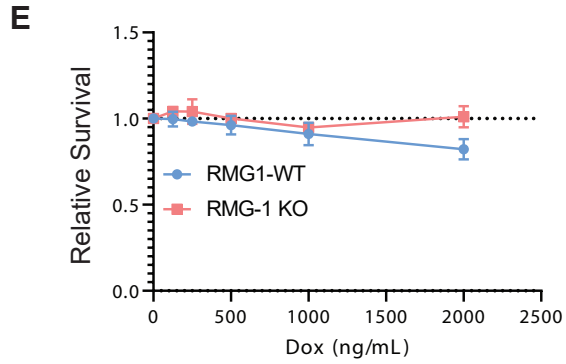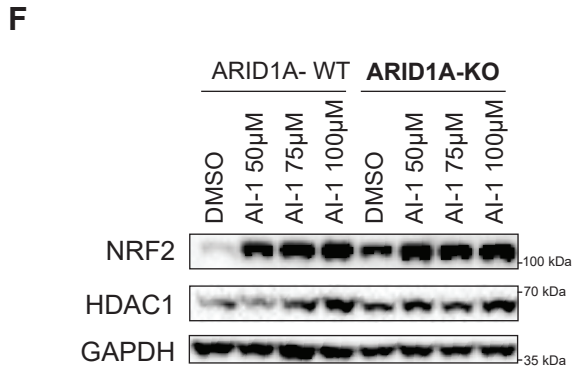

Supplement: Supplementary file 1 [file cancers-16-02949-s001.zip › Supplemental Figure S3_final.pdf]

Supplemental Figure S4

**A**

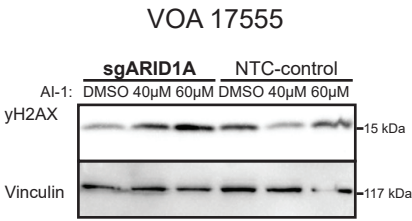

**B**

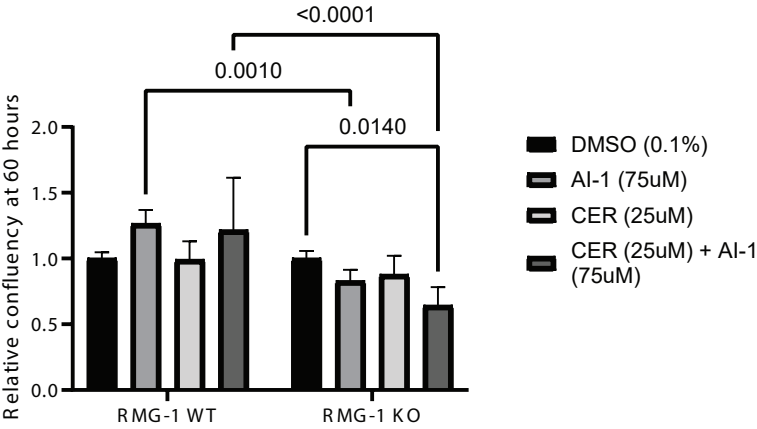

Supplement: Supplementary file 1 [file cancers-16-02949-s001.zip › Supplemental Figure S4_final.pdf]
